# Simultaneous analysis of the non-canonical amino acids norleucine and norvaline in biopharmaceutical-related fermentation processes by a new ultra-high performance liquid chromatography approach

**DOI:** 10.1007/s00726-013-1459-3

**Published:** 2013-01-11

**Authors:** Michael Biermann, Bettina Bardl, Sebastian Vollstädt, Julia Linnemann, Uwe Knüpfer, Guido Seidel, Uwe Horn

**Affiliations:** 1 Leibniz-Institute for Natural Product Research and Infection Biology (HKI), Beutenbergstrasse 11a, 07745 Jena, Germany; 2 Wacker Biotech GmbH, Hans-Knöll Strasse 3, 07745 Jena, Germany

**Keywords:** Recombinant antibody, Norleucine, Norvaline, Ultra-high performance liquid chromatography, Biopharmaceutical fermentation, *Escherichia coli*, Bioprocess design

## Abstract

In this study, a precise and reliable ultra-high performance liquid chromatography (UHPLC) method for the simultaneous determination of non-canonical (norvaline and norleucine) and standard amino acids (aspartic acid, glutamic acid, serine, histidine, glycine, threonine, arginine, tyrosine, methionine, valine, phenylalanine, isoleucine, leucine) in biopharmaceutical-related fermentation processes was established. After pre-column derivatization with *ortho*-phthaldialdehyde and 2-mercaptoethanol, the derivatives were separated on a *sub*-2 μm particle C_18_ reverse-phase column. Identification and quantification of amino acids were carried out by fluorescence detection. To test method feasibility on standard HPLC instruments, the assay was properly transferred to a core–shell particle C_18_ reverse-phase column. The limits of detection showed excellent sensitivity by values from 0.06 to 0.17 pmol per injection and limits of quantification between 0.19 and 0.89 pmol. In the present study, the newly established UHPLC method was applied to a recombinant antibody *Escherichia coli* fermentation process for the analysis of total free amino acids. We were able to specifically detect and quantify the unfavorable amino acids in such complex samples. Since we observed trace amounts of norvaline and norleucine during all fermentation phases, an obligatory process monitoring should be considered to improve quality of recombinant protein drugs in future.

## Introduction

Apart from the 20 standard amino acids, several non-canonical amino acids can be found in organisms. The amino acids norvaline and norleucine belong to a group of natural branched amino acid analogs synthesized by the biotechnological relevant bacterium *Escherichia coli* (*E. coli*) and other gram-negative species (Kisumi et al. 1976; Sugiura et al. 1981). Initially norvaline was described as a natural component of an antifungal peptide produced in *Bacillus subtilis* (Nandi and Sen 1953), while norleucine was found to be formed in leucine regulatory mutant variants of *Serratia marcescens* (Kisumi et al. 1976).

The biosynthesis of norleucine and norvaline by *E. coli* as byproducts of the branched chain amino acid biosynthesis occurs via the promiscuous enzymes of the (iso)-leucine pathway (Bogosian et al. 1989; Sycheva et al. 2007; Soini et al. 2008). Due to their broad range of keto acid substrate acceptance, the *leuABCD*-operon enzymes are proposed to facilitate a direct carbon chain elongation from the central intermediate pyruvate to the branched chain amino acid precursor 2-ketobutyrate which is synthesized from threonine under standard conditions. The following extension reactions of 2-ketobutyrate are catalyzed by the leucine biosynthesis enzymes isopropylmalate synthase (IPMS, *leuA* gene), isopropylmalate isomerase (IPMI, *leuCD* genes) and isopropylmalate dehydrogenase (IPMD, *leuB* gene). Enzymatic specificity of IPMS has not been investigated in *E. coli* yet but is known for a variety of substrates of the highly conserved homolog protein in *Salmonella typhimurium* (Kohlhaw et al. 1969) which can utilize, e.g., 2-ketobutyrate and pyruvate, for condensation with acetyl-CoA. Subsequent conversion of the intermediate compounds by IPMI and IPMD via this keto acid elongation pathway forms 2-ketovalerate and 2-ketocaproate. The last step in norleucine and norvaline biosynthesis consists of the transamination of 2-ketocaproate and 2-ketovalerate by aminotransferases IlvE, TyrA and AvtA.

The specific physiological conditions leading to formation of these modified amino acids in *E. coli* are not fully understood but experimental data suggest a strong connection to glucose overflow metabolism and pyruvate accumulation in fermentation processes (Soini et al. 2008). An additional proof for this hypothesis might be seen in the presence of norleucine and norvaline accumulation in *E. coli* knock-out mutants of the *ilvA* gene, which is responsible for 2-ketobutyrate synthesis from threonine (Sycheva et al. 2007).

Recently non-canonical amino acids have gained substantial interest when found incorporated into protein-based biopharmaceuticals produced by recombinant *E. coli* fermentation processes. Some examples of these unwanted misincorporations are the findings of norleucine in recombinant interleukin 2 (Lu et al. 1988; Tsai et al. 1988) and human brain derived factor (Sunasara et al. 1999) or norvaline in recombinant hemoglobin (Apostol et al. 1997). The incorporation of norleucine and norvaline occurs via misaminoacylation of the cognate tRNA during translation. Norleucine is known to be an isostructural analog of methionine, while norvaline is known to be an analog of leucine (Budisa et al. 1995). They can be mischarged to tRNA^met^ and tRNA^leu^ by aminoacyl-tRNA synthetases resulting in substitutions within the synthesized protein (Lu et al. 1988; Apostol et al. 1997; Reynolds et al. 2010). To insure the final quality of recombinant drugs, every modification of the active protein drug needs intensive analytical characterization according to the standards of regulatory authorities such as the US Food and Drug Administration and European Medicines Agency (Berkowitz et al. 2012; Ahmed et al. 2012). For this reason, early detection of non-canonical amino acids during process development in biopharmaceutical industry is required.

There is no universal technique for the detection and quantification of the mentioned amino acids in biological samples. The most common approaches for amino acid analysis include: liquid chromatography separation coupled with optical detection (Le Boucher et al. 1997; Joseph and Davies 1983; Fekkes 1996; Molnár-Perl 2005; Pappa-Louisi et al. 2007; Ilisz et al. 2012) and mass spectrometry-based detection methods coupled to prior separation by liquid or gas chromatography (Husek et al. 2008; Waterval et al. 2009; Armenta et al. 2010; Dettmer et al. 2012). However, these methods suffer from the limited number of covered amino acids, lack of separation due to slow mass transfer kinetics, ion suppression or expensive equipment (Kaspar et al. 2009).

In recent years, the ultra-high performance liquid chromatography (UHPLC) builds a new class of liquid chromatography with increased separation, sensitivity and speed (Wu and Clausen 2007; Fekete et al. 2012) of amino acid analysis. Either by the application of *sub*-2 μm material or core–shell particles, this approach achieves higher plate counts and column efficiency in comparison to conventional column materials (Guiochon and Gritti 2011; Gritti and Guiochon 2012).

In this study, a precise UHPLC method using a *sub*-2 μm C_18_ particle column was established for the simultaneous analysis of the non-canonical amino acids norleucine, norvaline and their corresponding isostructural compounds. Further method development implementing core–shell particle C_18_ material was carried out in order to allow the ultra-high performance assay on standard HPLC instrumentation. Both methods were also evaluated for crucial validation parameters. Capitalizing on the advantages of the core–shell method, application in recombinant antibody production was proved to be feasible. To our knowledge, this is the first report on OPA derivatization based simultaneous analysis of norleucine and norvaline by state of the art core–shell technique.

## Materials and methods

### Chemicals

Norleucine, norvaline, and Fluka A2161 amino acid reference solution for fluorescence detection, OPA and 2-mercaptoethanol were purchased from Sigma-Aldrich (Taufkirchen, Germany).

Hydrochloric acid, sodium hydroxide, di-sodium hydrogen phosphate, sodium dihydrogen phosphate dihydrate, methanol and tetrahydrofuran were from Merck (Darmstadt, Germany). Acetonitrile was purchased from VWR (Darmstadt, Germany). All chemicals were at highest available analysis grade. Water was purified by Milli-Q water purification system (Millipore, Bredford, USA).

### Instrumentation

A JASCO X-LC HPLC system (JASCO Corporation, Japan), containing two pumps, autosampler, intelligent column thermostat and fluorescence detector was used. For instrument control, data acquisition and analysis, JASCO Chrompass software was employed. Chromatographic separations were realized with Nucleodur C18 Gravity column (150 × 2.0 mm, 1.8 μm particle size), Macherey-Nagel, Düren, Germany) with column inlet filter Rheodyne (0.5 μm pore size, 3.0 mm I.D., Macherey-Nagel, Düren, Germany) and Sunshell C18 column (150 × 2.0 I.D. mm, 2.6 μm, dichrom, Marl, Germany).

### Chromatographic separation and quantification

In a reaction vial with conical insert, 30 μL OPA was added to 30 μL sample and mixed by autosampler unit. After 30 s reaction time, 1 μL of the solution was injected onto column. Mobile phase A was aqueous buffer (25 mM Na_2_HPO_4_/NaH_2_PO_4_, pH 7.2)/tetrahydrofuran (95:5, v/v) and mobile phase B was aqueous buffer (25 mM Na_2_HPO_4_/NaH_2_PO_4_, pH 7.2)/methanol/acetonitrile (50:35:15, v/v/v). The elution was facilitated by gradient program as follows: 0–0.6 min 10 % B, 0.6–9.0 min 50 % B, 9.0–48 min 60 % B, 48.0–51.0 min 100 % B, 51.0–56.0 min 100 % B, 56.0–57.0 min 10 % B, 57.0–59.9 min 10 % B. Temperature of the column oven was kept at 40 °C. The constant flow rate was 0.3 mL/min. Fluorescence detection and quantification was carried out by excitation wavelength 345 nm and emission wavelength 455 nm. The identification of sample peaks was realized by comparison of retention time with reference substances.

### Fermentation conditions and preparation of recombinant *E. coli* samples

Fermentation samples were obtained from cultivations of *E. coli* RV308 carrying plasmid p41-B10aP for VHH antibody fragment as described earlier (Horn et al. 1996; Habicht et al. 2007). Fermentation samples were prepared by quenching of fermentation broth containing medium and cells with −40 °C cold 60 % methanol and subsequent shock frozen in liquid nitrogen. Until analysis samples were stored at −80 °C. For amino acid analysis, samples were diluted to same biomass concentration with 0.9 % NaCl, followed by sonification for 10 min on ice. Removal of cell debris and deproteinization were carried out by centrifugation for 10 min (4 °C, 16,000×*g*) and ultrafiltration of supernatant using Amicon Ultra 3 kDa cut-off filter membranes.

## Results and discussion

### Chromatographic separation and fluorescence detection of norleucine, norvaline and standard amino acids

Based on the known advantages in sensitivity, selectivity and simplicity, the OPA derivatization (Roth 1971; Molnár-Perl 2005; Kaspar et al. 2009) was used for fully automated pre-column derivatization of reference amino acid solutions. We initially tested the reversed-phase HPLC method described by Kroemer (2005) as it reported good separation of the canonical branched chain amino acids leucine, valine, isoleucine. Although either norleucine or norvaline is separately used as traditional internal standards in analyses, this method failed in the simultaneous separation of norvaline, norleucine and their corresponding closely eluting amino acids valine and leucine. The obtained chromatograms showed no peak separation of these highly hydrophobic and isostructural amino acids (data not shown).

As a consequence, we chose a *sub*-2 μm C_18_ particle column (Nucleodur C18 Gravity) together with a smaller inner diameter of 2.1 mm to further increase separation efficiency of the method. To circumvent the compromising effect of a decreased column diameter, the number of theoretical plates was increased by keeping the column length at a dimension of 150 mm. The existing gradient protocol was optimized for UHPLC and applied to a reference solution.

**Fig. 1:**
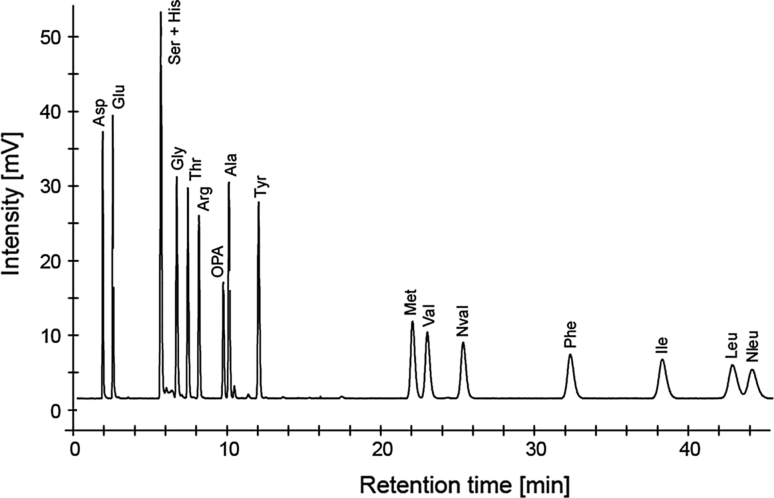
Representative core–shell UHPLC chromatogram of OPA derivatives of norleucine, norvaline and classical amino acids in reference solution obtained by fluorescence detection (each 12.5 μM)

All obtained reference chromatograms showed clear baseline resolution and symmetric peak shape for the non-canonical amino acids norvaline, norleucine and their proteinogenic isostructural relatives valine, leucine, isoleucine and methionine (data not shown). Following on from that, we were aiming to overcome the limitation of *sub*-2 μm particles to special equipment caused by high column back pressure (Fekete et al. 2012; Song et al. 2012a, b) of about 700 bar in our case respectively. Therefore, we directly transferred chromatographic conditions to the application of a 2.6-μm core–shell C_18_ column (Sunshell C18) with the same dimensions (150 mm × 2.1 mm). The obtained separation data were comparable to those from the *sub*-2 μm particle column (Fig. 1) showing equal resolution properties.

Emphasizing the enhanced performance of the hereby newly established method, the indirect resolution of amino acid derivatives that differ in hydrophobicity by only one methyl group (e.g., methionine/norleucine) was facilitated. Furthermore, the results show resolution of isostructural amino acids in both tested column materials.

### Analytical method validation

To evaluate the method, we determined the following validation parameters: linearity range, limits of detection (LOD), limits of quantification (LOQ), repeatability of retention time and intra-day precision (Table 1). The detection of OPA derivatives at 455 nm indicated linearity in the performed calibration range of 2.5–12.5 pmol (per 1 μL injection volume) with correlation coefficients of >0.99 for each analyte. LOD and LOQ were calculated as signal-to-noise ratios of 3 and 10, respectively. The resulting sensitivities (Table 1) were stated as pmol per injection, hence related to the actual on-column amounts of OPA derivatives.

**Table 1:** Validation parameters (*I*) of the core–shell UHPLC method for the determination of non-canonical and classical amino acids in biopharmaceutical fermentation processes

Amino acid	*R*_*t*_ (min)	Repeatability of *R*_*t*_ RSD (%)^a^	LOD (pmol)^b^	LOQ (pmol)^c^	Precision RSD (%)^d^
Asp	1.66	0.22	0.17	0.50	3.34
Glu	2.32	0.21	0.27	0.80	2.60
Ser/His	5.46	n.d.	n.d.	n.d.	2.19
Gly	6.48	0.10	0.08	0.25	4.77
Thr	7.21	0.09	0.14	0.41	5.55
Arg	7.93	0.08	0.10	0.29	2.42
Ala	9.90	0.08	0.33	0.98	1.50
Tyr	11.88	0.08	0.06	0.19	1.71
Met	22.01	0.17	0.08	0.24	2.22
Val	22.98	0.15	0.15	0.44	3.49
Nval	25.80	0.15	0.14	0.42	5.03
Phe	32.46	0.19	0.13	0.40	2.41
Ile	38.47	0.19	0.17	0.50	3.51
Leu	43.09	0.21	0.14	0.42	2.64
Nleu	45.10	0.22	0.10	0.31	3.18

^a^RSD, relative standard deviation based on five runs of reference solution (5 pmol per 1 μL injection volume) within 1 day

^b^Signal/noise ratio = 3

^c^Signal/noise ratio = 10

^d^RSD, relative standard deviation based on five runs of fermentation sample solution spiked with reference solution (5 pmol per 1 μL injection volume) within 1 day

The LOD for all OPA amino acids ranged from from 0.06 to 0.17 pmol per injection, LOQ were between 0.19 and 0.89 pmol. Comparing the new method to current OPA related studies (Pereira et al. 2008; Kaspar et al. 2009; Arrieta and Prats-Moya 2012) including analysis of complex biological matrices, it was possible to achieve a significant increase in sensitivity of chromatographic results. Overall sensitivity parameters of the method were also comparable to recent reports on *sub*-2 μm particle column (Mayer et al. 2010) and core–shell (Song et al. 2012a, b) amino acid analysis using different derivatization procedures and detection options. The obtained data show an overall improvement of detection sensitivity from μM to the nM range, which is a magnitude lower then LOD’s for OPA derivative analysis reported earlier (Wan et al. 2000).

### Simultaneous analysis of non-canonical amino acids in recombinant antibody fermentation

**Fig. 2:**
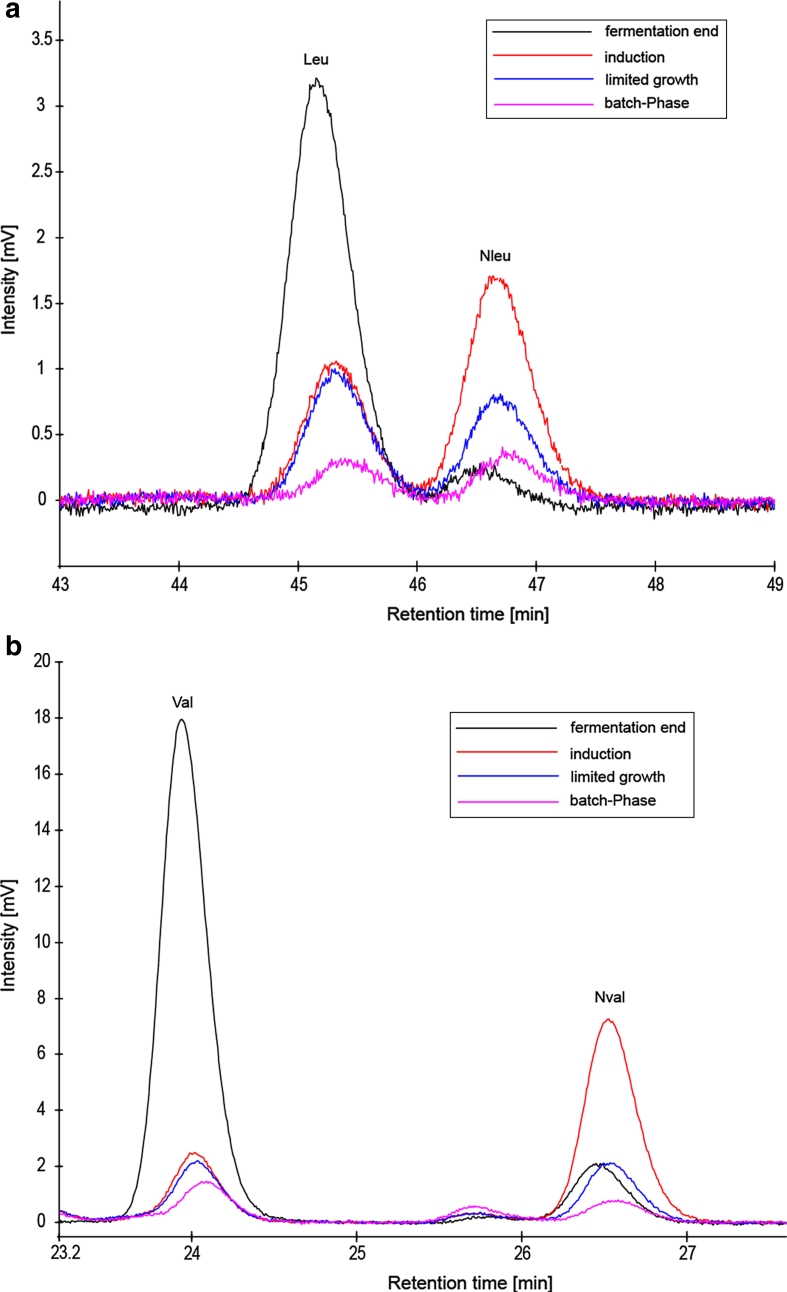
Chromatograms of OPA derivatives of leucine, norleucine (**a**), valine and norvaline (**b**) obtained from *E. coli* antibody fermentation. *Peaks* show different time points of *E. coli* cultivation

To investigate practical utility for the determination of non-proteinogenic amino acids in biopharmaceutical relevant processes, the method was applied to recombinant antibody fermentation samples of *E. coli* at different time points. The chromatograms shown in Fig. 2 were obtained from *E. coli* fermentations producing the recombinant camelid antibody domain B10 (Habicht et al. 2007).

Trace levels of norleucine and norvaline with concentrations of 0.66 and 0.92 μM were detected (Table 2) in early phase of the fermentation (batch phase). The values for both amino acids increased during glucose-limited growth up to 4.47 μM (Nleu) and 9.42 μM (Nval). Upon induction of the recombinant model antibody for 6 h, a significant decrease of norvaline and norleucine to a concentration of 2.60 and 0.44 μM was observed. This supports the argument of incorporation of these amino acids into recombinant proteins after induction (Sunasara et al. 1999; Apostol et al. 1997). The corresponding free pools of leucine and valine increased from the point of antibody production which might be due to the amino acid depletion caused by recombinant protein production (Bogosian et al. 1989).

**Table 2:** Concentrations of non-canonical amino acids and isomers in recombinant *E. coli* fermentation samples

	Nleu (μM)	Nval (μM)	Leu (μM)	Val (μM)
Batch phase	0.6	0.92	0.52	1.79
Limited growth	1.1	2.43	2.29	2.35
Induction	4.47	9.42	2.44	2.99
Fermentation end	0.44	2.60	8.44	23.93

The data shed new light into the occurrence of non-canonical amino acids since this is the first report on physiological free Nval or Nleu during balanced growth of *E. coli*. Biosynthesis of these compounds was proposed to be restricted to induction of recombinant gene expression (Apostol et al. 1997) or metabolic imbalance (Soini et al. 2008). Since our new method provides a significantly improved sensitivity, it seems to be plausible. A possible reason for this finding could be the higher sensitivity of the new method.

The results further indicate that this method can be applied for the simultaneous monitoring of low level concentrations of norleucine and norvaline in complex biological samples.

## Conclusion

The present novel approach allows the simultaneous, sensitive and precise detection and quantification of the non-canonical amino acids norleucine and norvaline. In addition, their canonical isostructural compounds leucine, isoleucine, valine, methionine and eight other proteinogenic amino acids can be efficiently resolved and quantified. By the analysis of recombinant *E. coli* antibody fermentation, the method was proved to be applicable to complex biological samples. No expensive instrumentation or time-consuming derivatization procedures are needed for assay performance. Taken together, this method provides a powerful tool in biopharmaceutical process analysis and further an improvement of protein based drug development.

## Abbreviations

*E. coli*: *Escherichia coli*
IPMS: Isopropylmalate synthase
IPMD: Isopropylmalate dehydrogenase
IPMI: Isopropylmalate isomerase
Nleu: Norleucine
Nval: Norvaline
OPA: *Ortho*-phthaldialdehyde
UHPLC: Ultra-high performance liquid chromatography
